# Recruiting pregnant smokers for a placebo-randomised controlled trial of nicotine replacement therapy

**DOI:** 10.1186/1472-6963-4-29

**Published:** 2004-11-01

**Authors:** Tim Coleman, Marilyn Antoniak, John Britton, Jim Thornton, Sarah Lewis, Kim Watts

**Affiliations:** 1 Division of Primary Care, University of Nottingham, Queen's University Medical Centre, Nottingham NG7 2UH England; 2 Division of Respiratory Medicine, University of Nottingham, Nottingham City Hospital NHS Trust, Hucknall Road Nottingham NG5 1PB England; 3 Division of Epidemiology and Public Health, University of Nottingham, Queen's University Medical Centre, Nottingham NG7 2UH England; 4 Division of Obstetrics, Gynaecology and Child Health, University of Nottingham, Nottingham City Hospital NHS Trust, Hucknall Road Nottingham NG5 1PB England; 5 Academic Division of Midwifery, University of Nottingham, Nottingham City Hospital NHS Trust, Hucknall Road NOTTINGHAM NG5 1PB England

## Abstract

**Background:**

Smoking in pregnancy is a public health problem and effective methods for reducing this are required. Although nicotine replacement therapy (NRT) is effective for smoking cessation in non-pregnant people, there is no direct evidence concerning its effectiveness in pregnancy. Despite this, clinical guidelines recommend the cautious use of NRT during pregnancy. Randomised controlled trials are needed to determine the safety and efficacy of NRT when used by pregnant women for smoking cessation, but the feasibility of recruiting women to such trials is unknown. Consequently, in this study we aimed to determine i) the feasibility of recruiting women to a RCT of NRT in pregnancy as they attend hospital antenatal ultrasound examinations, ii) the proportion of such women who are eligible for and interested in trial enrolment and iii) research staff perceptions of how one method of trial recruitment could be improved.

**Methods:**

During a one month period, all women attending for antenatal ultrasound examination in an English teaching hospital were asked to complete a questionnaire which determined their eligibility to enrol in a proposed placebo controlled randomised trial investigating the effectiveness of NRT in pregnancy. Women who were eligible to participate were asked whether they would do so and those who accepted enrolment were offered an appointment with a smoking cessation advisor.

**Results:**

Over 99% (851/858) of women agreed to complete a questionnaire about smoking habits whilst waiting for ultrasound examinations. 10.3% (88/851) of women attending for antenatal ultrasound fitted eligibility criteria for a proposed RCT of NRT in pregnancy, but only 3.6% [(31/851), 95% CI, 2.4 to 4.9%] indicated on the questionnaire that they would like to take part in a study involving randomisation to placebo or active patches. Researchers offered trial enrolment to 26 of these 31 women and 96% (25) accepted. Staff recruiting women believed that trial recruitment would be maximised if women attending the ultrasound department knew about trial recruitment before attending and greater staff resources were made available for this. It was also perceived that women generally under-reported the amount they smoked on questionnaires completed whilst waiting in ultrasound department areas.

**Conclusions:**

It is feasible to recruit women for a trial of NRT in pregnancy as they wait for antenatal ultrasound examinations. Using similar recruitment methods, researchers can expect to recruit between 24 and 49 women per 1000 approached.

## Background

Smoking harms unborn children causing intra-uterine growth restriction, preterm birth, miscarriage and perinatal death [1,2]. Despite this over a quarter of pregnant women in the UK smoke [3] and although pregnancy motivates a minority of women to stop smoking for at least part of their pregnancy, most start again after delivery [3]. Reducing smoking in pregnancy is, therefore, a health priority and effective methods for promoting smoking cessation in pregnancy are needed. Behavioural support is effective for smoking cessation in pregnancy, [4] but drug treatment is not generally used because of concerns that this may harm the fetus [5]. Outside of pregnancy, however, drug therapy can be effective and nicotine replacement therapy (NRT) increases quit rates of smoking cessation interventions it accompanies by 1.5 to 2-fold [6]. Unfortunately, the efficacy and safety of NRT in pregnancy are unknown.

Two trials conducted in pregnant women have shown NRT patches to have no greater effect on smoking cessation than placebo [7,8]. but neither trial has been large enough to be definitive. Additionally, nicotine is metabolised more quickly in pregnancy [9] and this could reduce the efficacy of NRT, as conventional doses will provide less nicotine substitution. Consequently, for NRT to be effective in pregnancy, higher doses might be needed but this raises questions of safety because nicotine is a recognised fetal toxin [10] and higher doses could increase the risk of any fetal damage occurring. Any such risk is difficult to quantify, though, because there is little human-subject evidence on the effects of nicotine (or NRT) in pregnancy and most evidence about the impact of nicotine in pregnancy is derived from animal studies [10]. In humans, nicotine is known to increase maternal blood pressure and heart rate but to have lesser effects on the fetal heart rate and these changes are less pronounced than those caused by smoking [10]. More encouragingly, the one completed trial of NRT in pregnancy [7] found babies born to women in the NRT treatment group had significantly higher birth weight than those treated with placebo, suggesting that intrauterine growth restriction caused by smoking is not attributable to nicotine. Further evidence on the safety of nicotine in pregnancy would help to inform clinicians' decisions about prescribing NRT in pregnancy.

Despite uncertainties about the effects of NRT in pregnancy, there is an expert consensus that NRT use is safer than smoking [11] which is reflected in clinical guidelines [5,12] and other advice to clinicians [13]. NRT use in pregnancy is, therefore, being encouraged in the absence of any direct evidence for it's effectiveness or safety and placebo-controlled RCTs are needed to resolve these issues. In pregnancy, women are generally advised to avoid unnecessary drugs [14] and it is likely, therefore, that women's reluctance to use drugs (including NRT, which contains a known fetal toxin [10]) might hinder recruitment to RCTs of NRT in pregnancy. Consequently, this paper describes a pilot study which aimed to determine: i) the feasibility of recruiting women to an RCT of NRT in pregnancy as they attended hospital antenatal ultrasound examinations, ii) the proportion of pregnant women attending such clinics who are eligible for enrolment and interested in participation in such a trial and iii) research staff perceptions of how one method of recruitment could be improved for use in a definitive RCT.

## Methods

Ethical approval was granted by the Nottingham Research Ethics Committee 2. For one month, one of three research assistants (RAs) was based in the Nottingham City Hospital antenatal ultrasound department for all scanning sessions. All women attending for ultrasound scans were approached by an RA and asked to fill in a self-completion questionnaire (see appendix) which asked their number of weeks gestation and whether or not they smoked at all. Respondents who answered that they smoked cigarettes or tobacco were then asked a series of questions about their smoking behaviour to ascertain whether or not they satisfied eligibility criteria for a proposed RCT (see Figure 2). Those smokers who were at an appropriate gestation, who smoked heavily enough for entry to the RCT and who were interested in stopping smoking completed the whole questionnaire. Before the final questionnaire item, they read brief information about the use of nicotine replacement therapy in pregnancy (which was on the questionnaire). The final item then asked women whether or not they would like to take part in a study which investigated the effectiveness of NRT in pregnancy. Those who did were subsequently offered enrolment into the study by the RA and their responses were noted. The enrolment offer included an explanation that no randomised study of NRT was currently taking place but that this was possible in the near future (which, at that time, the authors believed to be true). Women who agreed to enrolment were aware that they might participate in a study that would involve them being randomised to either active or inactive patches and, although nicotine was potentially harmful, NRT use was believed to be safer than smoking. RAs arranged appointments with the local NHS smoking cessation service [15] for those women who accepted the offer of enrolment and, if they attended these, women received counselling from midwives who specialised in smoking cessation counselling for pregnant women.

Enrolment criteria for the proposed trial (Figure 2) were constructed to ensure that only women who were addicted to nicotine were likely to be enrolled in the study. It was important to prevent any woman from entering a trial if it were possible that she might receive more nicotine via NRT dispensed in the trial than she otherwise would have received by smoking [11]. The antenatal ultrasound clinic setting was used because almost all pregnant women in the UK have an ultrasound scan performed between the end of the first and the middle of the second trimester of pregnancy. By recruiting in this setting, we were able to systematically approach all pregnant women residing in the local area who were at an appropriate stage of gestation and who were likely to deliver in the same hospital.

When the pilot was finished, the views of RAs and collaborating staff from the local NHS smoking cessation service were sought at a meeting. We asked their opinions of the utility of our method for detecting smokers (i.e. the questionnaire) and the practicality of recruitment within the ultrasound clinic setting. Where staff had criticisms, they were encouraged to suggest improvements in procedures. Key points made during this meeting were noted and issues described in the results section are those on which there was consensus.

### Sample size and data analysis

We knew from routinely-collected clinic statistics that over 800 women would attend for antenatal ultrasound scans in a four week period and around 200 of these (25%) would smoke. We hypothesised that 20% of smokers (i.e. approximately 40 or 5% of clinic attenders) would agree to trial randomisation. We believed this a reasonable assumption because in a survey of women attending ante-natal clinics, Ussher found that, amongst women who smoked 10 or more cigarettes daily and who were interested in stopping smoking; 56% were also interested in using NRT [16]. Consequently, if our survey was conducted for one month, we calculated that would be able to detect the proportion of clinic attenders agreeing to randomisation with 95% CIs of 1–2%.

All data was entered into an SPSS database and quantitative findings are expressed as simple proportions of all clinic attenders, with 95% CI for the principal outcome.

## Results

**Figure 1:**
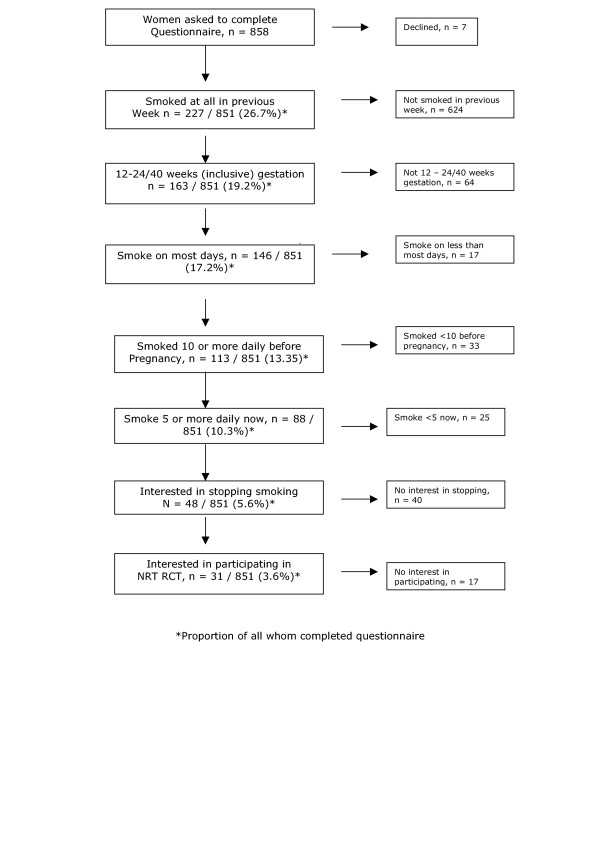
Flow Chart of Study Participation

Figure 1 shows that 99.3% of women attending for antenatal ultrasound imaging during the study period consented to complete a questionnaire. Of questionnaire respondents, around 27% had smoked in the last week, but only 163 of these were within the appropriate gestation range for trial entry. The diagram shows that although 17% of attenders admitted to smoking on "at least most days", this number fell to around 13% who admitted smoking 10 cigarettes daily before they were pregnant and it fell further to 10% who admitted smoking both 10 cigarettes daily before pregnancy and 5 currently. Of these heavier smokers, 5.6% were interested in stopping smoking. Finally, 3.6% [(31) 95%CI, 2.4 to 4.9%] of women attending for ultrasound both met trial entry criteria as measured on the questionnaire and were interested in participation in a study involving NRT. RAs offered study enrolment to 26 of these 31 women and 25 (96%) accepted. The remaining 5 women left the ultrasound clinic before RAs could discuss participation with them. 21 women who agreed to the enrolment offer attended at least one appointment with the local NHS smoking cessation in pregnancy advisor.

### Perceptions of research and smoking cessation service staff involved in pilot

There was consensus amongst RAs and NHS smoking cessation service staff that, if the following problems with study procedures were remedied, the proportion of women interested in trial participation who actually enrolled should increase:

• A number of women were interested in study participation but did not have sufficient time to discuss this with the RA in the clinic. These women might have been able to make time to discuss the study if they had known about the likelihood of being approached before attending the ultrasound department.

• On occasions, RAs failed to follow up women who had expressed an interest in study participation because they were busy with other duties (e.g. discussing enrolment with another woman).

• A number of women who had indicated on their questionnaire that they were interested in participation left the clinic before the RA could discuss enrolment with them. The pilot study did not have the resources to contact these women afterwards.

• Some women reported heavier smoking to smoking cessation advisors than they recorded on questionnaires. This apparent under-reporting of smoking behaviour may have resulted in some women who were actually eligible for study participation, not being offered study enrolment.

## Discussion

We have demonstrated that it is feasible to recruit women to a trial of NRT in pregnancy as they attend antenatal ultrasound appointments. Although almost all women consented to complete a questionnaire enquiring about their smoking habits, only 10% of these were eligible to enrol in an RCT and less than 4% appeared likely to participate in one.

Our study provides useful, original information for researchers planning trials of NRT in pregnancy. The two published trials of NRT use in pregnancy give no information about numbers of women were asked to participate but declined. One trial took two years to enrol 250 pregnant women [7] and the other recruited 30 women, but the time taken to achieve this is not reported [8]. To determine the level of interest in using NRT amongst pregnant smokers, Ussher and West conducted a telephone survey of women who had reported themselves to be smokers during a "booking" appointment (i.e. their initial antenatal appointment) [16]. They found that around one half of women who admitted to smoking at their antenatal "booking" appointment (95/177) were interested in receiving help with stopping smoking, and of those who admitted to smoking 10 or more cigarettes daily, 56% were interested in using NRT. This survey, however, was conducted amongst an ante-natal clinic population with a smoking prevalence of around 12%, which is much lower than the national average, so the generalisability of study findings to other areas is questionable. Additionally, it gave no indication of the numbers of women who might agree to randomisation which included the possibility of being allocated a placebo treatment. Nevertheless, our findings are not necessarily inconsistent with the London antenatal clinic survey. We found that approximately 35% (31/88) of women currently smoking five cigarettes daily (who also admitted to smoking 10 cigarettes daily before pregnancy) were interested in trial participation.

**Figure 2:**
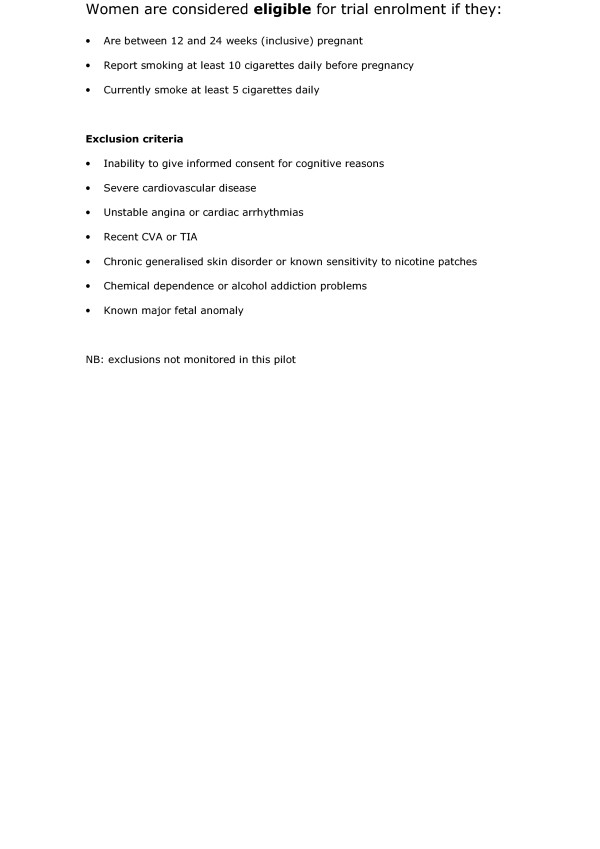
Eligibility criteria for proposed RCT

Our findings compliment those of Ussher's survey by providing information about likely participation rates for any randomised controlled trial. Extrapolating our figure of 3.6% indicates that approximately 36 (95%CI, 24 to 49) women could be recruited annually to a RCT of NRT in pregnancy from a maternity hospital with 1000 births/pregnancies each year. Researchers planning trials of NRT in pregnancy can use this recruitment rate to inform the planning of studies. Trialists should note, however, that we have measured agreement to be randomised rather than actual randomisation. Additionally, we did not measure the numbers of exclusions amongst women who were interested in participation. Figure 2, however, lists exclusion criteria for a RCT of NRT in pregnancy that the authors have proposed and consideration of these suggest that few pregnant women agreeing to enrolment would be excluded from using NRT on medical grounds. Finally, pilot study findings are likely to be most applicable to countries which have policies on the use of NRT in pregnancy that are similar to those employed in the UK: the proportion of women agreeing to randomisation would probably be lower in any health care system in which health professionals generally advised pregnant women against using NRT.

Some modifications to the recruitment method we used might be needed to maximise trial recruitment rates. Recruitment from antenatal ultrasound waiting areas might be increased if women were sent information about the likelihood of being approached to join any trial before they attended (e.g. sending information out with appointments). Additionally, providing enough resources to ensure that a member of trial staff is always present to ask patients to join a trial is important. This would overcome the problem of missing potentially interested women when the researcher responsible for recruiting is busy enrolling other potential participants. Any recruitment method should also allow for the fact that some women who are interested in participating will not be able to wait in the clinic to discuss this further. Recruitment would, therefore, be enhanced if enough resources were provided to allow research staff to make contact with these women later, visiting them at home if necessary.

The observation by smoking cessation service staff that some women admitted smoking more heavily to them than they admitted on questionnaires is consistent with the literature. Aveyard investigated the relationship between urinary cotinine levels and reported numbers of cigarettes smoked during pregnancy and concluded that, at booking, some pregnant women probably minimise the numbers of cigarettes that they report smoking [17]. Similarly, Owen and McNeil's investigation of salivary cotinine levels in pregnancy indicated that a small number of pregnant women actually conceal the fact that they smoke [18]. These research findings probably reflect the fact that, in the UK, there is a social stigma attached to smoking in pregnancy which is likely to bias questionnaire respondents against an honest disclosure of their smoking habit. Some women in our pilot may have been happier to disclose the full extent of their smoking to a supportive health professional than they were to a questionnaire distributed by a RA in an out patient waiting area.

Trialists could reduce the impact that under-reporting of heaviness of smoking might have on trial recruitment by avoiding the use of questionnaires to quantify smoking habits. Questionnaires used in recruitment to trials of NRT in pregnancy might be more effective if they merely ask women to disclose whether or not they smoke and if they are interested in using NRT to stop. All who smoke and wish to stop using NRT could then be invited for further discussion of trial entry with a researcher or health professional and during this disclosure a more accurate assessment of heaviness of smoking might be obtained.

## Conclusions

We have demonstrated that it is feasible to recruit pregnant women to a trial of nicotine replacement therapy in pregnancy in the antenatal ultrasound clinics. Using similar recruitment methods between 24 and 49 pregnant women per 1000 approached are likely to consent to participation in an RCT. RCT(s) of NRT in pregnancy are required and this data will help researchers planning such trials to determine the resources required to answer important questions concerning the effectiveness and safety of NRT in pregnancy.

## Competing interests

TC has been paid for speaking at a conference by GlaxoSmithKline (GSK); he has also done consultancy work on one occasion for Pharmacia (now Pfizer). JB has been reimbursed by Glaxo Wellcome (now GSK) for attending 2 international conferences, has received a speaker honorarium from Glaxo Wellcome, has been the principal investigator in a clinical trial of nicotine replacement therapy funded by Pharmacia and has undertaken consultancy work for Novartis. All companies manufacture nicotine replacement products but none were involved in this study. All other authors have no conflicts of interest.

## Authors' contributions

All authors were involved in various stages of study design. MA liaised with the NHS Stop Smoking and hospital antenatal services, collected data and supervised others in this. TC wrote the paper and all authors commented on drafts and approved final text.

## Pre-publication history

The pre-publication history for this paper can be accessed here:
